# Chromosome Replication in *Escherichia coli*: Life on the Scales

**DOI:** 10.3390/life2040286

**Published:** 2012-10-26

**Authors:** Vic Norris, Patrick Amar

**Affiliations:** 1 Theoretical Biology Unit, EA 3829, Department of Biology, University of Rouen, 76821, Mont Saint Aignan, France; 2 Laboratoire de Recherche en Informatique, Université Paris-Sud, and INRIA Saclay – Ile de France, AMIB Project, Orsay, France; E-Mail: pa@lri.fr

**Keywords:** DNA replication, cell division, growth rate control, ion condensation, water, evolution

## Abstract

At all levels of Life, systems evolve on the 'scales of equilibria'. At the level of bacteria, the individual cell must favor one of two opposing strategies and either take risks to grow or avoid risks to survive. It has been proposed in the Dualism hypothesis that the growth and survival strategies depend on non-equilibrium and equilibrium hyperstructures, respectively. It has been further proposed that the cell cycle itself is the way cells manage to balance the ratios of these types of hyperstructure so as to achieve the compromise solution of living on the two scales. Here, we attempt to re-interpret a major event, the initiation of chromosome replication in *Escherichia coli*, in the light of scales of equilibria. This entails thinking in terms of hyperstructures as responsible for intensity sensing and quantity sensing and how this sensing might help explain the role of the DnaA protein in initiation of replication. We outline experiments and an automaton approach to the cell cycle that should test and refine the scales concept.

## 1. Introduction

There are, apparently, two major classes of sins—those of commission and those of omission. In general, scientists seek to avoid the former class by refraining, for example, from venturing into unfamiliar disciplines where they risk talking nonsense. The problem with this strategy is that its partisans may fall foul of the latter class by omitting to look in another discipline where the solution to a problem within their own discipline is to be found. One remedy is for some of the community to indulge in clearly flagged speculation. Such speculation is particularly important in the case of fundamental problems such as how cells manage to generate coherent, reproducible phenotypes from the vast number of combinations of constituents available to them. For example, if the phenotype of *Escherichia coli* were determined solely by expressed genes, the number of phenotypes might be in the hyper-astronomical range of 2^4000^ combinations of these genes [1,2]. Another fundamental problem is the nature of the mechanism that controls the cell cycle of bacteria. These two problems are generally believed to be unrelated. The fact that they have resisted satisfactory solutions despite decades of intense research activity suggests that our thoughts are trapped in a local minimum in idea space and justifies an energetic speculation to help them escape from it.

What form should such speculation take? Benford, a physicist and an author of science fiction, which includes the remarkable *Timescape*, has proposed that there is "a link between the science I practise, and the fiction I deploy in order to think about the larger implications of my work, and of others." [3]. Useful speculation should be constrained by coherence—the novel things proposed should fit with one another and with other, known, things that are generally accepted. Such speculation should not be overly respectful of Occam's Razor since this carries the danger of over-simplification; a hypothesis should be, to cite a paraphrased phrase ('Einstein's Razor'): "as simple as possible, but not more simple". It should not be constrained by either discipline boundaries or the unfinished, controversial nature of the subject (such as water structure). It does not even need to correct to be useful: a speculation that is wrong can serve as a stepping stone to one that is right, much as a scaffold allows construction of an arch that supports an edifice. Whatever form it takes, good speculation should lead to a new and stimulating 'view from here' [4] and generate solutions not only to the original problem but also to other problems.

Some hypotheses, developed by those speculating at the interfaces between disciplines, have provided precious insights into living systems. This is the case, for example, of the hypothesis that life exists at the 'edge of chaos' which was developed at the interface between mathematics and biology as a solution to the problem of generating coherent, reproducible phenotypes [1,5,6]; in this hypothesis, regulatory systems evolve such that the connectivity between the regulatory elements is neither too little (which would give a 'frozen', unvarying, phenotype) nor too much (which would give an unpredictable phenotype). Speculation at the interface between physical chemistry and biology is another powerful generator of hypotheses. Via such speculation, several of us have developed potentially unifying hypotheses based on concepts in non-equilibrium thermodynamics that may, we hope, contribute to solving several fundamental problems—or, at least, to giving a new view of them. These concepts include non-equilibrium structures, such as whirlpools, which exist due to a flow of energy through them, and equilibrium (or, more often, *quasi*-equilibrium) structures, such as salt crystals, which exist in the absence of flows of energy. These concepts also include ion condensation, in which, *in vitro* at least, ions can condense or decondense from macromolecular structures in an abrupt transition that resembles a phase shift; such behaviour depends on the valence of the ions, the charge density and topology of the structures, and the structure of water [7,8].

The generation of the phenotype and the regulation of the cell cycle are, we would argue, subjects that could benefit from bold speculation. Such speculation might start from the premise that living systems are often forced to make a choice: either to interact, take risks and grow or to shut themselves away, hunker down and survive. Living systems are also highly structured. Indeed, in our "ecosystems-first", origins-of-life scenario, the most fundamental of living systems—cells—were highly structured right from the start [9,10]. We have argued that this structuring takes the form of extensive macromolecular assemblies, termed hyperstructures, which are the descendants of the macromolecular aggregates, alias *composomes *[11], with which life began. In this hypothesis, interaction between molecules on the basis of molecular complementarity created composomes in which these molecules were preserved from degradation in a continuous flux of abiotic creation and destruction; populations of composomes then co-evolved as a prebiotic ecology with natural selection acting on the various composomal species leading, eventually, to the emergence of linear replication [9]. Evidence for hyperstructures in both eukaryotes [12] and prokaryotes is steadily accumulating [13,14,15,16]. In bacteria, hyperstructures can be partly described by their position on an equilibrium–non-equilibrium axis [13]. Equilibrium hyperstructures, which remain stable despite interruptions in the flow of energy or nutrients, allow cells to survive starvation and stresses whilst non-equilibrium hyperstructures, which may fall apart as a result of such interruptions, allow growth. Examples of equilibrium structures include DNA in a cholesteric state, which can form in bacteria in the absence of metabolic activity [17,18], crystalline assemblies of DNA and the Dps protein that form to protect DNA during stress conditions [19], cardiolipin-rich domains in the membrane [20,21], enzyme filaments in which the enzymes are inactive (EF-Tu is a putative example) [22,23], and ribosomal subunits bound to DNA [24]. Examples of non-equilibrium hyperstructures include those formed by *transertion* (the coupling between transcription, translation and the insertion of nascent proteins into and through membrane) and *transembly* (the coupling between transcription, translation and the assembly of the products into a higher level structure); the ribosomal hyperstructure, which forms in *E. coli* growing rapidly, is a prime example of the latter [25,26,27]. The existence of these types of hyperstructures helps, in part, explain how cells overcome one of the major constraints on their evolution, namely that they must be able to both grow in heaven and survive in hell. This explanation is that a bacterial population comprises cells with different combinations of non-equilibrium and equilibrium hyperstructures which create the phenotypes [28]. This gives rise to another question—how do cells balance these types of hyperstructures? Put differently and in a way that is independent of the level of the system, this question boils down to 'How do living systems exist on the scales of equilibria?

At the level of cells, possible answers to this question involve the cell cycle itself as outlined in the Dualism hypothesis [28]. The first answer is that cells have been selected to sense the intensity with which certain non-equilibrium hyperstructures work. Consider, for example, the implication of the density of transcribing RNA polymerases per unit DNA being limited; this means that if the chromosome is not replicated, growth will eventually also be limited. A selective advantage might therefore be conferred on a cell that sensed when transcription risked becoming limiting and used this information to initiate chromosome replication. The second answer is that cells have been selected to avoid excessive accumulation of equilibrium structures. Consider, for example, an enzyme that could be catalytically active within a non-equilibrium hyperstructure and be catalytically inactive within an equilibrium hyperstructure (where it might have a structural role). Excessive accumulation of this enzyme in the inactive form would limit growth. A selective advantage might therefore be conferred on a cell that had learnt to sense the quantity of equilibrium hyperstructures and to use this information to dissociate them and to convert their constituent enzymes to the active form. In the Dualism hypothesis, these intensity-sensing and quantity-sensing processes drive the cell cycle so as to obtain different daughter cells in which different ratios of non-equilibrium to equilibrium hyperstructures (NE/E) provide evolutionary advantageous compromises between, for example, the catalysis needed for growth and the support needed for survival. As the basis of cell cycle signaling by hyperstructures that controls this life on the scales of equilibria, the Dualism hypothesis invokes potential mechanisms that include ion condensation and water structure.

If the scales of equilibria approach is really fundamental, it should offer a unifying framework for understanding the cell cycle of organisms as different as *Homo sapiens *and *E. coli*—and perhaps further still—from E.c. to E.T. Here, we re-examine one of the best understood of all systems, the initiation of chromosome replication in *E. coli*, from the standpoint of life on the scales. We make experimental predictions and, given the value of related, automaton approaches to the cell cycle [29], we also suggest a simulation approach based on genetic algorithms and a hybrid automaton, HSIM [30].

## 2. Problems with Current Explanations of Initiation Control

During state-state growth, a bacterium like *E. coli* [31] must initiate chromosome replication at the same rate as its mass increases since failure to do so would lead to cells either filling up with DNA or lacking it. Over forty years ago, two classes of models began to vie with one another to explain the initiation of chromosome replication (a very different type of model received less attention [32]). The first class, which had been used successfully for plasmids, invoked a repressor acting at the origin of replication, *oriC*, that was diluted by growth to allow initiation every mass doubling [33]. The second class invoked an activator of replication that accumulated to a critical level to trigger initiation during the mass doubling; this class of model benefited in particular from an analysis of data [34,35] that suggested that the bacterium initiated replication at a constant mass—the initiation mass—regardless of growth rate [36]. The DnaA protein became identified with this activator [37]. Unfortunately for simple models based on the dilution of a repressor or the accumulation of an activator with respect to an origin of replication, it was found possible to create minichromosomes (small plasmids that replicated using *oriC*) [38]. Worse, the *E. coli* cell cycle was found to be unperturbed by the presence of scores of these minichromosomes that even replicated in synchrony with the chromosome itself [39]. An adapted version of the model of DnaA as the activator was proposed in which DnaA first binds to many high affinity sites throughout the chromosome before finally binding to lower affinity sites in *oriC* to trigger initiation [40]; hence, the chromosome itself became part of the timing mechanism.

The original idea of the initiation mass came into question when it was found that the initiation mass varies at different growth rates and it was concluded that "the timing of initiation is not governed by a direct connection between mass accumulation and the molecule(s) determining initiation of replication" [41,42,43]; more recent studies using cell size mutants have led to the suggestion that the initiation mass in *E. coli* depends on the amount of DnaA rather than on its concentration and, moreover, that the initiation mass model must be different for *B. subtilis* [44]. Models for explaining initiation mass also have to contend with the decrease in the mass of DNA (but unaltered rates of growth and division) when *dnaAts* mutants are grown at semi-permissive temperatures, which gave rise to the idea that DnaA controls the number of initiations but not their timing [45]. Worse still, the initiation of replication of minichromosomes is apparently normal in a strain in which the chromosome itself replicates at random using the origin of replication of a plasmid [46]; this effectively disqualifies the chromosome itself from being the essential element in the timer. Most seriously of all, perhaps, the presence of an extra *oriC* in the *E. coli* chromosome, which results in two origins that function at the same time, has relatively little effect on the cell cycle [47,48].

Recent models for initiation have been based on the proportion of the DnaA 'initiator' protein in the ATP-DnaA form, which is active in initiation, or the ADP-DnaA form, which is not. There are several hypotheses to explain how the level of active DnaA might increase cyclically to trigger initiation [49]. Lipids have long been known to be involved in initiation [50] and one possibility is that anionic phospholipids might promote conversion from ADP-DnaA to ATP-DnaA *in vivo* as *in vitro* [51]. Another, complementary, possibility is that specific DNA sequences promote this conversion via the formation of homomultimers of the DnaA protein [52]. Following initiation, removal of DnaA may also be achieved by DnaA binding to sequences in the *datA* locus [53]. These proposed mechanisms do not answer the fundamental question of how an increase ATP-DnaA levels would be timed to trigger initiation.

## 3. The Dualism Hypothesis

Many, perhaps all, living systems are obliged, at some time in their existence, to balance the requirement to grow with the requirement to survive. Without growth, the proportion of the niche that they fill would decrease (even if they reproduce). Without survival, the game is over (even if conditions permit growth). The fundamental problem is the requirements for growth and survival are opposed. Growth cannot occur without non-equilibrium structures. Survival, particularly in conditions of deprivation, cannot occur without equilibrium (more exactly, quasi-equilibrium) structures. The fundamental question is how do living systems reconcile these contradictory demands. To put it in a picturesque way, how do they manage to live on the scales of equilibria?

The archetypal, terrestrial living system is the cell. Cells contain both non-equilibrium and equilibrium structures or, more precisely, hyperstructures. We propose that the answer for cells to the fundamental question of 'grow or survive?' involves them balancing their non-equilibrium and equilibrium constituents. Previously, we have argued that differentiation is built into the cell cycle so as to give one daughter cell equipped for growth and the other for survival; this stems from the logic of the regulation of cellular circuitry based on local positive feedback (a gene that is being expressed may have a greater chance of continuing to be expressed) and global negative feedback (genes may be competing with one another for expression) [54]. Some evidence of this propensity to differentiate may be found in the distribution of genes on the strands with the genes needed for growth tending to be on one strand and those for resistance to stresses tending to be on the other strand [55].

**Figure 1:**
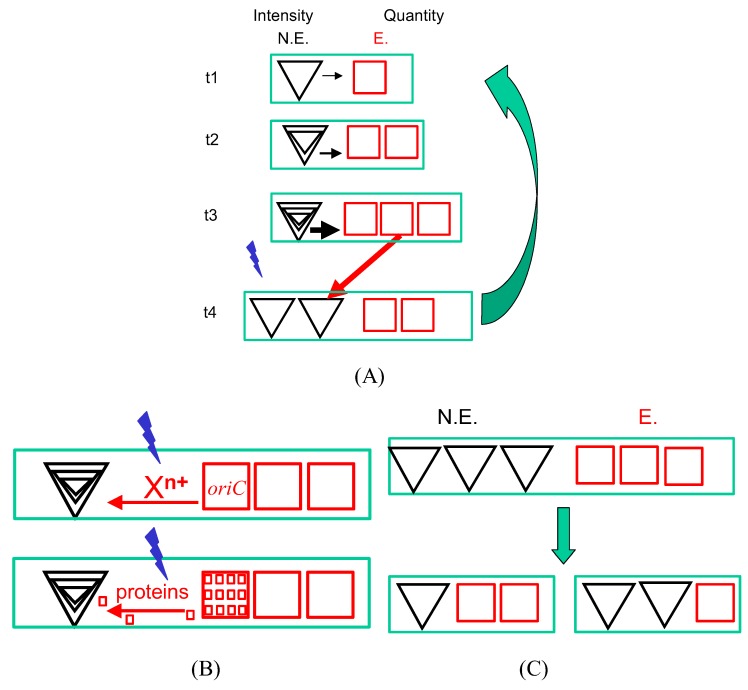
Life on the scales. A/ The essence of the cell cycle. A new cycle starts at the time t1 as a result of two processes—intensity changes and quantity changes—affecting non-equilibrium hyperstructures (N.E., black triangles) and equilibrium hyperstructures (E., red squares). The growing bacterium is represented by the green rectangles. The quantity of equilibrium hyperstructures, relative to non-equilibrium ones increases (red arrows); in parallel, the non-equilibrium hyperstructures generate equilibrium material with an increasing intensity (t1 to t3). At time t3 in the cell cycle, a signal is emitted (blue lightning) that leads to some of the equilibrium material being transformed into non-equilibrium hyperstructures and that triggers initiation of chromosome replication from origins which were in an equilibrium state. B/ The nature of the signal. The blue lightning represents the event that occurs at a threshold. Top rectangle: the blue lightning represents attaining the charge parameter needed for ion decondensation in the case of the equilibrium hyperstructures and attaining the charge parameter needed for ion condensation in the case of the non-equilibrium hyperstructures; the consequent movement of cations is shown. Bottom rectangle: the change in ion distribution leads to a redistribution of macromolecules involved in transcription and translation from equlibrium to non-equilibrium hyperstructures. C/ Different daughters. The daughter cells have different ratios of NE:E so giving them initially different phenotypes that equip them to either grow rapidly or resist stress.

In the subsequent Dualism hypothesis, fleshed out here, we continue to explore how the cell cycle results in cells achieving the right ratio—or range of ratios—of non-equilibrium to equilibrium structures (the NE:E ratio). This entails the cell sensing the NE:E ratio, which occurs in two ways (Figure 1):

(1). Intensity sensing. The intensity of use of non-equilibrium hyperstructures is sensed. This intensity reflects the availability of energy (e.g., ATP and GTP) and the availability of nutrients (e.g., amino acids plus ATP, GTP, UTP and CTP). In the case where the intensity sensing is performed by a ribosomal hyperstructure (for references to its morphology in different conditions see [56]), this non-equilibrium hyperstructure serves as an integrator or as an AND gate insofar as the state of the hyperstructure reflects the metabolic state of the cell at that *particular moment in time*. When this intensity is sufficiently high for growth to risk being limited, one or more signals are emitted by these hyperstructures. These signals may be of very different natures (see below).

(2). Quantity sensing. The quantity of equilibrium hyperstructures is sensed. In the case where the quantity sensing is performed by a filament of inactive enzymes, the quantity of this equilibrium hyperstructure increases over time and the state of this hyperstructure reflects the history of the cell. When this quantity is sufficient for daughter cells to be generated, a signal is emitted. It is of likely evolutionary advantage that a quantity sensing mechanism exists to ensure that before a cell embarks on a new cell cycle it has sufficient quantities of membrane, energy and nucleotides to get it through to the end (cell division) even if there is a disaster.

Both intensity and quantity signals are needed for a cell to begin a new cycle and initiate the production of daughter cells. The physical nature of these signals may be different in different living systems.

## 4. Mechanisms

The two classes of cell cycle signals are generated by two different sets of mechanisms. Here, we give examples of candidate mechanisms.

### 4.1. Intensity Sensing

It can be of evolutionary advantage for a cell to grow (and reproduce) as rapidly as possible in favourable conditions. Such growth involves RNA polymerases transcribing genes to make mRNA, and ribosomes translating mRNA to make proteins. There is a finite minimum distance between two RNA polymerases transcribing a gene [57] (and between two ribosomes translating mRNA [58]). This means that in the absence of the DNA replication part of the cell cycle, the density of RNA polymerases per unit DNA eventually reaches a maximum (*i.e.*, when the polymerases cannot get any closer). From then on, growth can no longer be exponential. A selective pressure therefore exists in favour of cells that replicate their DNA before it becomes limiting. The solution would be *transcriptional sensing* which has been proposed for bacteria [59] but which is equally applicable to eukaryotes. The signal leading to DNA replication—indeed, the actual trigger itself—could take one or more diverse forms including multivalent ions, enzymes and metabolites (see Figure 1). The creation (and perhaps the nature of the signal(s) itself) might involve coupled transcription-translation (transertion) of a proteolipid hyperstructure [59], coupled transcription-translation-assembly (transembly) of a ribosomal hyperstructure with a particular topology [27,60], a giant elastic or electromagnetic oscillation [61], changes in water structure and ion condensation [28], and changes related to supercoiling [27]. A related solution—but which would not involve DNA directly—would be via *metabolic sensing* in which the dynamics of cytoskeletal hyperstructures (and hence cell cycle progress) would depend on the activity of metabolic enzymes such that, for example, an enzyme that is catalyzing its reaction has a greater affinity for the replication and/or cytoskeletal hyperstructures and helps to stabilize/destabilize them [62]. A good example of metabolic sensing occurs in the control over cell size in *Bacillus subtilis* where assembly of the tubulin-like FtsZ into an effective division structure is inhibited by interaction between FtsZ and the glucosyltransferase UgtP; this interaction is greater when UDP-glucose levels are high, as during growth in rich medium, leading to bigger cells [63]. In studying the relationship between metabolism and signaling *in silico*, we have found that a judicious choice of different sets of rate constants, equilibrium constants and initial conditions produces a variety of kinetics of metabolite concentrations and enzyme associations that could serve as signals [64].

### 4.2. Quantity Sensing

Even in conditions that are generally favorable for growth, there will be times when the non-equilibrium hyperstructures can either only grow linearly or just maintain themselves or shrink, whilst the equilibrium hyperstructures continue to accumulate. As the cell grows in the run-up to the key cell cycle event, new material (macromolecules, molecules and inorganic ions) comes from the non-equilibrium hyperstructures feeding both themselves and the equilibrium hyperstructures. An example of this material may be certain ribosomal subunits which can participate both in the non-equilibrium ribosomal hyperstructure and, conceivably, in equilibrium hyperstructures that also contain DNA [24,65]; note that inactive ribosomes can exist as clusters [66,67]. Another example may be EF-Tu [22,23] which can be active in translation but perhaps—evidence is needed—inactive as a cytoskeletal filament. In one version of the Dualism hypothesis, this progressive addition of material is accompanied by other changes such as a redistribution of water structures and changes in the topologies and sizes of both the equilibrium and non-equilibrium hyperstructures (note that resonant frequencies of oscillations are another possibility). This redistribution of material and water structures eventually results in the charge parameters of the equilibrium and non-equilibrium hyperstructures reaching a threshold that leads to ion decondensation and condensation, respectively [28,68]. This threshold is dependent on the topology and composition of the hyperstructures, such as the alignment of positive or negative charges, rather than, say, the average level of charge. The changes that occur at this threshold time the cell cycle.

## 5. The Specific Case of DnaA in the *E. coli *Cell Cycle

### 5.1. DnaA Background

DnaA, a protein found in many bacteria, is commonly believed to be responsible for the initiation of chromosome replication in bacteria [49,69,70,71]. In the Beginning, however, DnaA was not present and a more fundamental mechanism operated. DnaA would therefore have evolved as a veneer on this mechanism. If so, it may prove possible to interpret DnaA action in the framework of Dualism. This we now attempt.

DnaA binds with different affinities to sites in the origin of replication, *oriC*, and elsewhere on the chromosome [72,73]. The synergistic binding to *oriC* sites results in the unwinding of an AT-rich region and the opening of the double helix that constitutes the first step in initiation [74]. DnaA binds ATP and ADP, can form oligomers, and interacts with lipids. But what controls DnaA? One regulatory mechanism affects the availability of DnaA: substantial proportion of DnaA binds to the *datA *locus [53].

Another regulatory mechanism for controlling DnaA depends on the direct activation of DnaA [75] which results from it binding and hydrolysing ATP (only the ATP-DnaA form is active in initiation [76]). Sufficient activation for initiation requires the reactivation of existing ADP-DnaA since *de novo* synthesis of DnaA is not enough [77]. This activation can result from the binding of DnaA to either the membrane [78] or DnaA-reactivating sequences, DARS1 and DARS2, on the chromosome [52].

Another factor to take into account in the control over DnaA is that fluorescent DnaA-GFP hybrids bind not only to *oriC* and *datA* to give foci or clusters but also elsewhere in the chromosome to give a helix [79,80,81]. It should be noted here and elsewhere that fusions with fluorescent probes such as GFP can lead to artifactual localization of the protein under study [82,83]. It might be argued that such fusions may sometimes increase slightly the degree of a self-association that occurs naturally. It has been shown recently that MreB, rather than forming a helix on its own, forms a proteolipid domain with fluid phospholipids [154] and these patches move independently along a helical path [84,85]. It may turn out that the distribution of DnaA and MreB are both controlled by helical lipid domains created by transertion [86].

How can these characteristics of DnaA be accommodated by the Dualism hypothesis in the context of intensity sensing and quantity sensing (see Table 1 for a summary)?

**Table 1:** Characteristics of the DnaA protein (and factors that may affect it) can be classed as suggestive of its role in either intensity sensing or quantity sensing (see text for details).

Intensity Sensing	Quantity Sensing
**Superhelicity.** Increased superhelicity during growth might release DnaA from DnaA binding at sites on the chromosome [87].	**Cardiolipin**. CL detaches DnaA from spiral hyperstructure (CL detaches DnaA from DNA) [87]. CL domains are also important in division [90].
**ATP levels**. If several DnaA are bound to DARS they can be recharged with ATP (ATP^4-^ condenses before ADP^3-^) [52].	**Nucleoid Associated Proteins**. NAPs include HU, H-NS, Fis, IHF, StpA and Dps; each binds to 100s of specific sites + every 100 bp. IHF, HU and Dps interact with DnaA [91,92]. IHF for example also binds to oriC, helps DnaA bind to datA and binds itself to datA (IHF deletion delays initiation) [93]. H-NS is in a hyperstructure [16] and H-NS deletion delays initiation
**Size/topology of ATP synthase transertion** **hyperstructure** [88]. atp genes are close to *oriC* and hence to a *datA*-IHF-DnaA hyperstructure.	
**Central Carbon Metabolism**. Acetyl phosphate level is transduced by a DnaA hyperstructure (*dnaA46(ts)* suppression by *pta* (phosphate acetyletransferase) or *ackA* (acetate kinase) [89]	

### 5.2. Membrane

The membrane is strongly implicated in the behaviour of DnaA. Acidic phospholipids in a fluid phase dissociate ADP or ATP from DnaA so as to convert, in the presence of ATP, inactive ADP-DnaA to the active ATP-DnaA and allow DNA replication *in vitro* [51,94,95]; in this, cardiolipin is particularly effective whilst phosphatidylethanolamine and other lipids are either ineffective or inhibitory [96,97,98]. Membrane domains have long been proposed to regulate the bacterial cell cycle [99,100,101] and there is now considerable evidence for domains enriched in cardiolipin at the poles and between segregating chromosomes [2,90,102,103]. There is also complementary evidence for the existence of domains of high microviscosity, resulting from transertion, around the chromosomes [104,105] and even for an increase during the cell cycle in the level of cardiolipin and other lipids [104,105,106,107]. In the context of Dualism, cardiolipin-rich, fluid domains might correspond to equilibrium structures such that their accumulation would correspond to *quantity sensing*. More specifically, during the build-up to initiation, transertion hyperstructures around the chromosome become increasingly active (*i.e.*, with more transcription, more translation and more insertion of nascent proteins into membrane) generating and releasing cardiolipin to accumulate elsewhere until a threshold for reactivation of DnaA is attained. This threshold quantity would be the one at which there is sufficient membrane to allow successful completion of a new round of the cell cycle. Note too that this threshold might also depend on domains of cardiolipin acting as a proton trap to sense the energy status of the cell [108].

### 5.3. DnaA Boxes and DatA

The binding of DnaA to its sites plays a central role in its activity. There are around 350 DnaA proteins per origin (but up to a total of 2500 DnaA in a fast growing cell where there are also many origins) [109] and these proteins can bind with differing affinities to around 300 DnaA boxes in the chromosome [110]. These boxes are found in regions that include *oriC *and *datA*, which are able to bind 45 and 370 DnaA proteins with apparent dissociation constants of 8*.*6 × 10^−9^ M and 1*.*7 × 10^−8^ M respectively [111]. DnaA binding to *datA* is consistent with a titration role in initiation because such binding appears to delay initiation or prevent hyper-initiation [81,112,113].

In this titration role, *datA* does not fit readily into the class of equilibrium structures that are subject to quantity sensing (if it were to perform quantity sensing, we might expect, for example, the number of copies of *datA* to accumulate—which they do not—and, in accumulating, progressively bind only ADP-DnaA to give an increased ratio of free ATP-DnaA:ADP-DnaA); unless, of course, *datA* is physically part of an *equilibrium* origin hyperstructure—certainly, it does seem close to *oriC* [71,81]. In this case, the equilibrium material that accumulates (and that is sensed by the origin hyperstructure in its equilibrium state) might include some of the Nucleoid-Associated Proteins or *NAPs*, which are central to the organization, replication, segregation, repair and expression of bacterial chromosomes, and which are essential if a new cell cycle is to be completed successfully. The NAPs include HU, H-NS, Fis, Integration Host Factor (IHF), StpA and Dps, each of which binds to several hundred specific sites throughout the chromosomes and which together bind non-specifically to the chromosome at an average of one NAP per hundred base pairs [114,115,116,117,118]. Several NAPs, including IHF, HU and Dps, associate with DnaA [91,92]. IHF, for example, binds to many sites on the chromosome [16], and, in bending DNA, often acts together with other NAPs and transcription factors; for example, IHF interacts with DnaA and Fis to initiate replication via IHF binding to a site in *oriC* to stimulate unwinding [119,120]. This makes IHF a good candidate for playing a role in quantity sensing. It is therefore significant that IHF binds to *datA* and that this binding increases the number of DnaA proteins that bind *datA* [93]. It will be apparent to the reader at this stage that the DNA-binding protein best-placed for the lead role in a quantity sensing hyperstructure—perhaps as the first amongst equals—is DnaA itself.

Instead of *datA* belonging to the class of quantity sensing, equilibrium hyperstructures, could it belong to the class of intensity sensing, non-equilibrium hyperstructures? Here, one is looking for evidence, for example, that the superhelicity of the *datA* region increases during the build-up to initiation and that this releases DnaA. We know of no direct evidence for this (although DnaA binding to DNA is affected by superhelicity, see below). It is, of course, conceivable that a *datA*-IHF-DnaA hyperstructure is involved in *intensity sensing*. Such a role could be related to the proximity to *oriC* of the genes encoding subunits of ATP synthase which themselves are likely to create an ATP synthase transertion hyperstructure with a size and topology that should change with continued growth (and also to the distribution of gyrase sites as discussed in [27]); in the case in which spiral lipid domains result from transertion, it is significant that disruption of the domains of MreB plus fluid phospholipids affects the activity of LacY and the F_1_F_0_ ATP synthase [154].

### 5.4. DARSs

DARS1 and DARS2 [52], in the chromosome are directly responsible for part of the conversion of ADP-DnaA into ATP-DnaA needed for initiation. DARS1 (located between *bioD* and *uvrB*) and DARS2 (located between *ygpD* and *mutH*) contain three similarly organised DnaA-binding sites and, in the case of assembly of DnaA proteins into homomultimers on DARS1, the resultant interactions between them reduce the affinity of DnaA for ADP. Since the reactivation of DnaA could be observed *in vitro* with a fragment DARS1, supercoiling is not needed. This excludes one way in which DARSs could form part of an intensity sensing, non-equilibrium hyperstructure. There may, however, be something else related to a quantity sensing, equilibrium hyperstructure. To follow the reasoning, a reminder about ion condensation may be useful here.

The condensation of positive ions such as potassium, magnesium, calcium and polyamines onto negatively charged linear polymers (such as DNA, RNA and certain protein filaments) and onto anionic membrane domains leads to these counterions being delocalised and diffusing in the near region in intimate contact with the polymers and membranes [121,122,123]. Ion condensation onto linear polymers occurs when the dimensionless charge parameter, ξ, reaches a critical value where ξ = βe/(4πε_0_εkT), β is the linear charge density (defined as β = z_p_e/b with z_p_ the valence of the charges on the polymer, b the distance in nm between these fixed charges and e the elementary charge), ε_0 _is the permittivity of vacuum, ε is the relative dielectric constant of the medium, k the Boltzmann constant and T the absolute temperature. In water at 25 °C, ξ = 0.71/b for monovalent fixed charges on the polymer. When ξ exceeds a critical value of 1/z, counterions of valence z condense onto the polymer. As counterions condense onto such polymers they lower the linear charge density. Counterions condense onto the polymer in the order of their valence, for example, first all the tetravalent ions condense (providing the charge parameter remains high enough), then the trivalent, then the divalent and finally the monovalent ions condense. The dielectric constant of water, on which the charge parameter depends, must vary with water structure, which remains an important area of uncertainty.

Ion condensation onto charged polymers, such as DNA or protein filaments, is related to temperature so that it might occur at 30 °C but not at 0 °C [7,8]. It may therefore be significant that a PCR-generated fragment containing DARS1 promoted dissociation *in vitro* of ADP-DnaA at 30 °C but not at 0 °C [52]; the binding of DnaA itself to DARS1 and DARS2 is not affected by these temperatures [52]. As mentioned above, such condensation is related to both the charge of the counterion and the density of charge along the polymer: at the critical charge parameter of the polymer, for example, all the quadrivalent ions such as ATP^4^^−^ would condense onto the polymer before the trivalent ones such as ADP^3^^−^; moreover, the linear structure of the polymer is important. It may therefore also be significant that ATP can recharge DARS-bound DnaA and that, for this to happen, several copies of DnaA must bind to the DARS [52]; this might be explained by the condensation of ATP^4^^−^ onto a DARS-bound DnaA structure. In the Dualism approach, it is speculated that quantity sensing may take the form of positively charged counterions condensing onto equilibrium hyperstructures until the threshold is attained at which counterions decondense from the origin region and the consequent repulsion of the phosphates leads to strand separation and initiation of replication [28,124,125]. If there is some substance to this speculation, it would not be surprising to see the same phenomenon recapitulated with the DARS in which they have evolved to become representative of ion condensation on quantity sensing, equilibrium structures. One might take it further and speculate that when the DARS cease to be equilibrium structures they cease to be able to reactivate DnaA. The speculation goes like this: DARS1 and DARS2 are located right next to genes that are expressed in response to DNA damage; if this were to result in a change in the conformation of the DARS (e.g., their becoming single-stranded), the critical charge parameter would be lowered, the condensation of ATP^4^^−^ might cease, ATP-DnaA levels drop and initiation be averted until the damaged DNA had been repaired.

### 5.5. DnaA Spirals and Foci

To return to intensity sensing, consider again the distribution of DnaA. The protein, is distributed at the membrane either as foci that colocalise with the origin of replication, *oriC* [81], or as a helical spiral [80] or as both (but for *B. subtilis* see [79]). Such hyperstructures may well contain acidic phospholipids such as cardiolipin [126]. In the following, we reason in terms of a linear polymer of DnaA rather than a cluster (or even a higher order spiral of clusters) because the role we propose for ion condensation is easier to explore for a linear polymer than for a two dimensional domain. The helical form of DnaA may—or may not—interact with both the numerous sites of DnaA on the chromosome and the membrane [80]. (Note here that the spiral is seen in the nucleoid regions and, even if it can be seen in inter-nucleoid regions, these regions may still contain DNA). If we suppose that the DnaA in this spiral is binding to both its sites on the chromosome and to membrane, there is an interesting possibility that this DnaA hyperstructure is involved in intensity sensing. This is because this spiral hyperstructure might well be responsive to the changes in superhelicity that can accompany growth [27] such that at a threshold superhelicity the spiral would be destabilized (e.g., partly unwound) and DnaA released from these sites ready to recharged and to participate in initiation; there is indeed evidence that DnaA in some circumstances can be detached from DNA by increased superhelicity [87]. The DnaA spiral would therefore constitute an intensity sensor. In fact, it might be even better than this: the DnaA spiral hyperstructure might act as a quantity sensor by responding to an increase in cardiolipin. This is because acidic phospholipids (such as cardiolipin) in a fluid phase can detach DnaA from DNA [87] and hence an increase in cardiolipin might also be sensed by the DnaA spiral hyperstructure and transduced into the signal for the initiation of replication.

### 5.6. H-NS

In the hyperstructure dynamics approach to the cell, the interactions between hyperstructures determine the phenotype. If, then, the spiral DnaA hyperstructure is indeed acting as a sensor, there should be interactions with other hyperstructures in addition to the origin hyperstructure. These might be expected to include the recently described H-NS hyperstructure, which regulates 5% of all *E. coli* genes and brings together its regulated operons into two or so clusters per chromosome [16]. Deletions of H-NS halved the time to replicate the chromosome and delayed initiation (insofar as there was a 50% increase in the mass at which initiation occurred) [127]. This would be consistent with the H-NS hyperstructure normally contributing to destabilizing the spiral DnaA hyperstructure. It should be noted that IHF, which does not form the same type of hyperstructure [16], has similar effects on the initiation mass and replication times [128].

### 5.7. Ribonucleotide Reductase

In the quantity sensing vein, a sensible cell would ensure that it had enough nucleotides before embarking on a new cell cycle. Deoxyribonucleotides can be generated from the ribonucleotides that constitute RNA (and from the ATP and GTP that make up the energy currency of the cell) by the enzyme ribonucleotide reductase which is located in the replication hyperstructure [129,130,131].

### 5.8. Central Carbon Metabolism (CCM)

The central carbon metabolism is the set of biochemical pathways responsible for the transport and oxidation of main carbon sources and, in *E. coli*, comprises the phosphotransferase system, glycolysis, gluconeogenesis, the pentose-monophosphate bypass with the Entner-Dudoroff pathway, Krebs cycle with the glyoxylate bypass and the respiratory chain. A relationship between the enzymes involved in CCM and the elongation step in DNA replication was first observed in *B. subtilis* [132]. This has now been extended to *E. coli* where it has been found that the temperature sensitivity of a strain containing the *dnaB(ts)* mutation could be suppressed by dysfunction of *pgi* or *pta*; *dnaE486(ts)* by dysfunction of *tktB*; *dnaG(ts)* by dysfunction of *gpmA*, *pta* or *ackA*; *dnaN159(ts)* by dysfunction of *pta* or *ackA* [133]. In particular, a full suppression of the thermosensitivity of *dnaA46(ts)* was observed in *pta* (encoding phosphate acetyltransferase) or *ackA* (encoding acetate kinase) double mutants. The acetyl phosphate level, which may be reduced in the Pta- and raised in the AckA^−^ contexts, is thought to act as a global signal in two-component systems linking metabolism with other systems [134]. Could this relationship between DnaA and Pta/AckA reflect intensity sensing? Suppose that free Pta/AckA were to bind DnaA. Then, if the formation of a FDS containing Pta and AckA (and perhaps other CCM enzymes too) were to depend on the intensity of functioning of these enzymes, the availability of ATP-DnaA might increase as the proportion of free Pta/AckA decreased. There is, however, no evidence for direct interaction between DnaA and CCM enzymes and other explanations are possible, as discussed by the authors [132,133].

### 5.9. Ribosomes

Interactions between DnaA and ribosomal protein L2 (or truncated L2) have been shown to inhibit initiation *in vitro* and it has been proposed that DnaA may act as a sensor coordinating initiation with cell growth [92]. However, interpreting this result in the context of Dualism is not straightforward. It is not in line with a ribosomal hyperstructure acting as an intensity sensor because, the prediction is that if DNA is becoming limiting, redundant ribosomes may start to accumulate, become inactive and perhaps release L2 (we are trying here to see past layers of sophisticated feedback controls) which should stimulate initiation. The opposite occurs. It is not in line with quantity sensing either because, again, the prediction is that if ribosomes accumulate as equilibrium structures, release of L2 should again stimulate initiation. Although no hypothesis should be required to explain all the data, Dualism might offer an explanation: the signal that results from the combination of intensity and quantity sensing and that initiates replication also then prevents further initiation (along the lines of the Regulatory Inactivation of DnaA in which, after initiation, the Hda protein plus DNA promote the hydrolysis of active ATP-DnaA to inactive ADP-DnaA [71]). So the prediction from Dualism would then be that in the conversion of equilibrium to non-equilibrium structures—and the consequent redeployment of ribosomes—L2 is released to contribute the inhibition of further initiations. Finally, given that L2 also interacts with a heat shock protein, HtpG, it is conceivable that its inhibition of DnaA is simply a response to stress rather than part of regulation in an unperturbed cycle [135].

Many levels of classical regulatory systems have been glossed over in this and other sections. One of the most important is growth rate control. In the rest of this subsection, we suggest how growth rate control might fit into our scenario. Growth rate control is about the major changes that occur in Enterobacteriaceae as they adapt to environments that differ in their nutritional richness. The mechanisms involved in growth rate control might therefore be expected to be involved in the changes that we propose occur during the cell cycle. As a result of growth rate control, Enterobacteriaceae change as the rate of balanced growth increases: they become larger, have more replication forks per chromosome and have an increased RNA:mass ratio (primarily because more ribosomes are formed per unit mass) [34,56,136,137,138]. This increase in ribosomes affects the size and topology of the ribosomal hyperstructure [26,56]. One of the major players in control rate control is (p)ppGpp [139]. pppGpp and ppGpp are phosphorylated derivatives of GTP and GDP, respectively, obtained when a ribosome engaged in translation encounters an uncharged tRNA [139]. (p)ppGpp is an important link in coupling nutrient availability with many aspects of bacterial physiology including growth rate control, adaptation, secondary metabolism, survival, persistence, motility, biofilm formation, development, competence, virulence and initiation of DNA replication and cell division.

In section 5.4, the phenomenon of ion condensation was described in terms of positive counterions condensing onto negative linear polymers: it can be applied equally well to negative counterions condensing onto positive linear polymers. In *E. coli*, high levels of (p)ppGpp inhibit growth and change gene expression to reduce unnecessary activities in non-growing cells whilst excessively low levels result in cells responding inadequately to nutritional stress [139]. In the context of a role for ion condensation in life on the scales of equilibria, we might expect (p)ppGpp to decrease the average NE/E ratio. This might occur, for example, by (p)ppGpp condensing onto arrangements of positively charged residues within equilibrium hyperstructures so as to stabilise them and/or (p)ppGpp condensing onto similar arrangements of positive charges with non-equilibrium hyperstructures so as to increase the maximum density of usage that can be attained. RelA is the enzyme in *E. coli* that catalyses the reaction generating (p)ppGpp. It has been proposed that RelA can form oligomers in which it is inactive whilst starvation for amino acids results in the RelA oligomers dissociating to generate active enzymes [140]. It has also been proposed that RelA associated with the ribosome is inactive and that RelA dissociates from the ribosome to become active [141]. Both propositions are consistent with RelA activity depending on it being part of either a non-equilibrium or an equilibrium hyperstructure (that could respond to the condensation of ions including (p)ppGpp itself). Finally, (p)ppGpp inhibits replication by interacting with the DNA primase, DnaG, which is a key constituent of the replication hyperstructure [142] as well as being a product of the intriguing Macromolecular Synthesis operon which also contains *rpoD *and *rpsU* (encoding key proteins in transcription and translation) and which might therefore form a transcription-translation hyperstructure onto which (p)ppGpp condenses.

## 6. Constitutive Stable Replication

If the modern *oriC*-DnaA system is a molecular veneer, it should be possible to return to an ancient, more fundamental system. Arguably, such a system has been revealed in *E. coli*. In the absence of *oriC* and DnaA, mutants defective in the RNAse, RnhA, are able to replicate the chromosome from five other origins, *oriK*s, in what is known as constitutive stable replication [143]. Like normal initiation, stable replication involves unwinding the DNA helix and making DNA-protein hyperstructures. Initiation is thought to begin at a R-loop (a structure of two strands of DNA and one of RNA); the one strand of RNA is annealed with its complementary strand of DNA, which causes the other strand of DNA to be displaced from the helix; the RNA for the R-loop is transcribed by RNA polymerase and RecA probably then forms or stabilizes the R-loop; the RNA in the R-loop is then extended by DNA polymerase I to form a D-loop [143,144]. The explanation for constitutive stable replication requiring RNase HI is that this enzyme preferentially degrades the RNA primers at sites other than *oriC* because DnaA protects the primer at *oriC *[143].

The challenge for the Dualism hypothesis is to explain stable replication in terms of non-equilibrium and equilibrium hyperstructures responsible for intensity sensing and quantity sensing. The probability of initiation at one of the *oriK* is presumably related to the number and activity of the RNA polymerases at the locus. This would correspond to the class of intensity sensing termed transcriptional sensing. The probability of R-loop formation is increased by the negative superhelicity produced by DNA gyrase which again constitutes a type of intensity sensing (see above) [143].

## 7. Predictions

Many experimental predictions based on Dualism have already been made [28]. They include:

### 7.1. Non-Equilibrium and Equilibrium Hyperstructures

The existence of two types of extended structures with different time scales might be tested by pulse-labelling bacteria with nutrients enriched with isotopes such as ^13^C, ^14^C and ^15^N and imaging using Secondary Ion Mass Spectrometry [145,146].

### 7.2. Ion Condensation

If the phenomenon of condensation underlies the recharging of DnaA with ATP by the DARS, this might be demonstrated by combining an *in vitro* system of differentially, isotope-labeled ATP and ADP (or ATP-DnaA and ADP-DnaA) and fragments containing DARS, with the technique of Combing Imaging by Secondary Ion Mass Spectrometry, CIS [147]. This would require testing for the effects of varying temperature, ion concentrations and even water structures.

### 7.3. Quantity Sensing

Runaway production of equilibrium hyperstructures should lead to initiation. Constructs might be used to induce the production of lipids and NAPs and the effects tested on the numbers of origins (e.g., using isotope-labeling and density gradient centrifugation or runout experiments, or indeed CIS). Such induction is also predicted to have effects on the distribution of DnaA (be it in spirals, patches, or spirals of patches).

### 7.4. Intensity Sensing

Induction of formation of transertion or transembly hyperstructures [28], via expression systems based on IPTG, arabinose, T7, *etc.* should lead to initiation and could be tested as in 7.3. above. Alterations in the DnaA hyperstructure should result from changes in superhelicity and, as in quantity sensing, from changes in the composition of the membrane.

### 7.5. Constitutive Stable Replication

It would be interesting to investigate quantity sensing in this context by, for example, exploring the relationships between constitutive stable replication and the accumulation and distribution of phospholipids such as cardiolipin and of NAPs, where one might expect the genes neighboring the *oriK*s to be related to lipids and DNA-binding proteins. It has been reported that minichromosomes cannot be replicated in conditions of constitutive stable replication [143,148], which opens up the possibility that they might be replicated if they were to contain the genes and DNA-binding sites needed for intensity and quantity sensing hyperstructures.

### 7.6. Simulation

In principle, hybrid automata such as HSIM [30] could simulate cells containing two sorts of molecule, black and red, that would assemble into non-equilibrium and equilibrium hyperstructures respectively. A third, blue, molecule or ion with an affinity for both types would then be responsible for the dialogue between the two types of hyperstructure; the numbers of this ion are proportional to the volume of the cell; the ion has an affinity for both hyperstructures. Movement of blue ions from the black non-equilibrium hyperstructures to the red equilibrium hyperstructures might then be achieved if the blue ions were to sense (via changes in affinities) the intensity of activity of the black hyperstructures and the quantity of the red hyperstructures. A fundamental question that might then be addressed is whether, in this competitive system, there are relationships between the parameters—and values of the parameters—that can generate a clear, precise signal.

## 8. Discussion

A fundamental characteristic of living systems is, we argue, their ability to balance the conflicting requirements of growth and survival. This entails them balancing the proportion of their mass in the form of two structures that exist on different time scales. In the case of bacteria, in the Dualism hypothesis, these two structures can be equated to non-equilibrium hyperstructures, responsible for growth, and to equilibrium hyperstructures, responsible for survival, and the cell cycle becomes the way to distribute them amongst daughter cells so as to produce a diverse population in which cells are equipped differently for growth and survival [28]. This is what we mean by 'Life on the scales of equilibria' (Figure 1). It has a thermodynamics flavor insofar as the signal to initiate a new cell cycle derives from a combination of intensity sensing and quantity sensing, echoing perhaps intensive and extensive variables [149]. In proposing the Dualism hypothesis, we have also argued that cells also use intensity and quantity sensing to confirm that they have enough resources to complete the cell cycle successfully before initiating it.

The task we have taken on here is to see whether the 'scales of equilibria' approach might serve as a unifying framework for the vast and diverse quantity of molecular information on the cell cycle. We have therefore examined one of the most intensely studied cell cycle events, initiation of chromosome replication in *E. coli*. We show how it may be possible to use the concepts of non-equilibrium, intensity sensing hyperstructures and equilibrium, quantity sensing hyperstructures to class the factors known to be important in the regulation of initiation. These factors include ATP-DnaA, ADP-DnaA, DARS, *datA*, anionic phospholipids, the NAPs and chromosome supercoiling. Other factors which might also have been incorporated include the possibility of metabolic sensing by hyperstructures [62,132] that include the replication hyperstructure [150] and the EF-Tu hyperstructure [23,151], as well as the possibility of a relationship between calcium concentrations and ATP levels [152]. We consider whether the scales of equilibria approach might be useful in interpreting constitutive stable replication, believed to correspond to the vestiges of an early replication system [143].

Ion condensation, decondensation and water structures might play the lead role in the dialogue between hyperstructures that, in the Dualism hypothesis, results in initiation of replication. In our re-examination of the literature on initiation of chromosome replication in *E. coli*, we find an intriguing clue implicating ion condensation in the activation of DnaA by DARS [52] and even *in vitro* evidence for a role for condensation in the case of another key player in the cell cycle, FtsZ [153]. That said, a role for ion condensation *in vivo* remains controversial. Finally, to persuade biologists that the scales of equilibria is a useful concept for understanding the cell cycle, simulation is needed to clarify the concept and experiments are needed to lend it support.

## 9. Conclusions

At many levels, living systems are obliged to find compromises between the evolutionary virtues of growth and survival. We have proposed in the Dualism hypothesis that this quasi-universal need for a compromise solution results in Life on the scales of equilibria. In the case of bacteria, we have further proposed that a solution is provided by the cell cycle itself and that this entails the bacterium integrating (1) an intensity sensing that gives information about the non-equilibrium hyperstructures required for growth and (2) a quantity sensing that gives information about the equilibrium hyperstructures required for survival. Here, we examine results in the literature about DnaA, the key protein in the initiation of chromosome replication, and interpret these results in terms of intensity and quantity sensing.
